# Supplementary material for: Knowledge, risk of infection, and vaccination status of hepatitis B virus among rural high school students in Nanumba North and South Districts of Ghana
Source: PLoS One. 2020 Apr 22;15(4):e0231930. doi: 10.1371/journal.pone.0231930 (PMC7176126; doi:10.1371/journal.pone.0231930)
Supplement: Attachment — Submitted filename: Knowledge and Vaccination Status of Hepatitis B-Reviewed.docx [file pone.0231930.s001.docx]

**Knowledge, Risk of Infection, and Vaccination Status of Hepatitis B Virus among Rural High School Students in the Nanumba North and Nanumba South Districts of Ghana**

**Awolu Adam^1,2^**

^1^School of Public Health, University of Health and Allied Sciences, Ho, Ghana

^2^Center for Health Literacy and Rural Health Promotion

P. O. Box GH1563, Accra, Ghana

Adam Fusheini^2,3^

^3^Department of Preventive and Social Medicine, Dunedin School of Medicine,

P.O. Box 56, 9054, Dunedin

University of Otago, Dunedin, New Zealand

**Abstract:**

**Background:** Hepatitis B is a viral infection that affects the liver and can lead to life threatening conditions including cirrhosis and liver cancer. Over a billion people are estimated to be infected globally with hepatitis B virus with over 240 million chronically infected. Sub-Saharan Africa is an endemic for HBV and an estimated 5-10 % of the population in the region is infected. Research on the knowledge and vaccination status of hepatitis B in rural communities in Ghana is lacking.

**Objectives:** The objectives of this proposed were to assess the hepatitis B virus knowledge, risk and vaccination status of high school students in two rural districts of the Northern region of Ghana

**Methods:** A cross sectional study of a random sample of 426 students from two senior high schools in the Nanumba North and South districts of the Northern region on hepatitis B knowledge and vaccination status was conducted. Descriptive statistics was used to analyze and present data on demographic and knowledge variables. Chi square was used to test hypotheses and multiple regression analysis was used to develop a model for predicting vaccination against HBV.

**Results:** The results of the study showed general awareness but not comprehensive knowledge of HBV among the rural high school students with a mean score of 18.4 ±SD=2.24 out of a total obtainable score of ___. Descriptive statistics also revealed that only 20% of 426 students ever tested for HBV and 96 (22.5%) vaccinated against HBV. Regression analysis showed that taking HBV test predicted vaccination against HBV infection (*P=0.000*) at 95% confidence interval..

**Conclusion:**

The results of this study has re-echoed the high prevalence of HBV in Ghana. The poor state of knowledge and high risk of HBV infection among young adults in rural communities have also been highlighted in the findings of this study. Vaccination against HBV infection was found to be low and consistent with other findings. Finally, HBV screening is shown to be significantly associated with vaccination against the virus, hence the need for national screening and vaccination programs.

Key Words: Hepatitis B, rural-Ghana, young adults, vaccination, screening.

**Introduction:**

Viral hepatitis B (HBV) infection is a serious health condition and constitutes one of the big public health challenges globally and especially in developing countries. The Center for Disease Control and Prevention (CDC) described hepatitis B as a liver infection caused by HBV which can be transmitted through blood, semen, and other bodily fluids, and from a mother to her newborn through birth [1]. Hepatitis B infection is a potential life threatening infection as it can lead to chronic and debilitating health conditions including cirrhosis, liver cancer and death [1,2]. The risk of developing chronic hepatitis B infection is depended on the age at which an individual is infected. For instance, the CDC estimated that 90% of infected infants will become chronically ill compared to 2-6% of those who are infected as adults [1].

Data on prevalence of HBV infections globally is scary but attention paid to the infection seems inadequate especially in the endemic regions of sub-Saharan Africa and Asia. There are differences in the global estimates of chronic HBV prevalence but all are pointing to a serious global picture and particularly sub-Sahara Africa and East Asia. For instance, the World Health Organization (WHO) estimated that in 2015, 257 million people were living with chronic hepatitis B infection which is defined as hepatitis B surface antigen positive and that the estimated deaths due to HBV infections were 887,000 globally [2]. Most of the HBV related deaths were from cirrhosis and hepatocellular carcinoma. Other estimates indicate that 2 billion people globally have been infected with HBV with more than 350 million developing chronic and lifelong infections [3]. In fact, all estimates of HBV prevalence show that HBV is more infectious than even HIV and those affected by HBV far outnumber those afflicted with HIV/AIDS since its peak in the 1980s. HBV and hepatitis C are root causes of liver cancer, leading to 1.34 million deaths every year [2].

Like HIV/AIDS, HBV is endemic in sub-Sahara Africa and East Asia, where societies are poverty afflicted and knowledge about the disease is lowest including Ghana. According to WHO, HBV prevalence is highest in the WHO Western Pacific Region and the WHO African Region, where 6.2% and 6.1% of the adult population is infected respectively [2]. Ghana is equally a HBV endemic country as a number of screening programs reported a high prevalence rate. For instance, researchers used Wondfo HBsAg test kit to test the prevalence of HBsAg among blood donors at the Tamale Teaching Hospital in Ghana and reported that those who donated blood were two groups; voluntary donors (576) and blood replacement donors (5,878) making up a total of 6,462 [4]. The researchers reported that 10.79% of voluntary donors and 11.59% of replacement donors were HBsAg positive [4]. This was just one institution and the rate is indicative of bigger problem as it relates to only health facility reports. Another screening program test among 1,500 pregnant women in the Eastern region of Ghana reported 10.6% hepatitis B prevalence among the participants with some variations in the districts covered [5]. Again, among 838 HIV positive persons in Ghana, researchers used rapid test kits to test prevalence of co-infection of HBV and HIV and reported overall prevalence of 16.7% [6]. More recently in 2019, researchers have reported 8.5% and 14.2% prevalence of chronic HBV and occult hepatitis respectively isolated from 305 dried blood spots collected in Ghana [7]. The above prevalence rates corroborate WHO’s report of 5-10% adult prevalence rates in sub-Sahara Africa.

Analysis of the HBV prevalence/incidence data from Ghana Health Service (GHS) district health information management systems (DHIMS2) shows a worrying trend. Organized or standardized data on hepatitis B was lacking until the implementation of DHIMS by GHS in 2010. DHIMS data on reported HBV infection is categorized into suspected cases, confirmed cases, and death resulting from HBV complications. The trend shows increasing incidence/prevalence in the country. For example, confirmed cases in 2012, 2013, and 2014 nationally as reported were 3508, 4419, and 7324 respectively [8]. Reported HBV related deaths for the three years were 164, 101, and 131 respectively [8]. It is worth noting that suspected cases were far more than confirmed cased in these years and also that the figures reported here were institutions-based reported cases only. There are also regional variations in both suspected and confirmed cases of HBV prevalence as well as HBV related deaths. Using the confirmed HBV infection cases for 2014 for instance, Ashanti, Brong Ahafo, Greater Accra, Northern, and Upper West regions had 918, 1200, 2155, 288, and 1124 cases respectively. It could therefore be inferred that HBV is endemic in Ghana.

The transmission of HBV from one person to another can be through unprotected sex, blood transfusion, mother to child transmission, and through other bodily fluids. Unprotected sex, blood transfusion and mother to child transmission have been reported to be the major routes of HBV infection in Ghana [4]. HBV therefore seems to have more transmission routes than many sexually transmitted diseases including HIV. However, mother to child transmission appears to be one of the greatest risks of HBV infection in children. Hepatitis B virus (HBV) infection in a pregnant woman poses a serious risk to her infant at birth. This is because without postexposure immunoprophylaxis, approximately 40% of infants born to HBV-infected mothers in the United States will develop chronic HBV infection, approximately one-fourth of whom will eventually die from chronic liver disease [9]. By age 6 months, there is risk of chronic HBV infection in 90% of infants in mothers with hepatitis B surface antigen (HBsAg) and hepatitis B e antigen if no immunoprophylaxis is done [10]. HBV has also been found as co-infection among people living with HIV in Ghana and other diseases. In a systematic review of studies on HIV/HBV co-infection conducted across most regions in Ghana, researchers found HIV/HBV con-infection rate of between 2.4% and 13.6% with a general co-infection rate of 13.6% [11]. In studying the etiology of viral hepatitis in 155 jaundiced patients in a teaching hospital in Ghana, researchers found that 54.2% of them tested positive for hepatitis B and 32.9% tested positive for hepatitis E [12].

The most effective and efficient way to prevent HBV infection in a population is through vaccination. Unfortunately, HBV awareness, access to screening, vaccination and treatment have remained poor in resource limited countries due to poverty, illiteracy and lack of political will [13]. In 2003, HBV vaccination was introduced for all newly born in Ghana and screening of pregnant women to prevent mother-child transmission is now ongoing. Prior to 2003, there was no national HBV vaccination and so all those born before 2003 had the greatest risk of mother-to-child transmission. However, the introduction of the immunization policy has resulted in less infections and lower prevalence among those who were born after the introduction of the HBV immunization policy. To evaluate the impact of the immunization policy on HBV prevalence in Cape Coast Municipality of the Central Region of Ghana, researchers sampled 501 school children in a pilot study and found that the prevalence of HBcAb was 2.6% and 6.1% among those born after the introduction of the policy and those born prior to the introduction of the policy [14]. That is significant differences and attestation that immunization of new born babies is critical to ending the HBV epidemic in Ghana.

There is currently no national HBV screening program for adult population and young people born before 2003. Only episodic screenings are done in the general population mainly by non-profit organizations. Educational programs are lacking in terms of HBV in Ghana coupled with inadequate attention or resource commitment. As result of poor attention, knowledge about HBV as well as HBV vaccine is poor in many developing countries. In examining knowledge, accessibility, and HBV vaccination status of 643 pregnant women at three levels of healthcare delivery in Ibadan, Nigeria, researchers reported that 76% of the pregnant women had very low levels of knowledge of HBV, 20% reported being screened, and only 9.7% vaccinated against HBV infection [13]. In the study, being a healthcare worker, attaining higher education, and seeking healthcare at tertiary levels increased the likelihood of improved knowledge, previous screening, and vaccination against HBV infection. Among 370 health workers sampled in Bahir City in Ethiopia, only 52% scored above average knowledge about HBV infection, 62% exhibited adequate knowledge about HBV vaccine, and only 5.4% completed three doses of HBV vaccination [15]. In a study of knowledge, perceptions, and practices about HBV among the general population of France, both HBV knowledge and vaccination were found to be poor [16]. Using a telephone interview, the researchers interviewed 9,014 individuals between ages 18 and 69 years and reported that compared to HIV, knowledge of HBV was poor and only 27.4% ever screened for HBV [16]. Numerous similar findings of poor HBV knowledge and low vaccination have been reported in other settings [17, 18, 19, 20]. Therefore, the poor knowledge and low vaccination rates seem pervasive globally especially in developing countries.

A few studies on HBV in Ghana have been analyzed but almost all the studies tested prevalence rates among some selected groups in the country or short screenings. Only a few of studies examined knowledge, attitudes and practices in relation to HBV. For instance, in a cross-sectional study of HBV knowledge, attitudes, and practices (KAP) among 175 healthcare workers at Suntreso Government Hospital, Ghana, researchers reported basic knowledge or awareness of HBV with age, occupation, and years of experience being significant predictors of KAP. Using qualitative methods, researchers examined perceptions and understanding of HBV in the Upper West region of Ghana and found ‘extremely low levels of knowledge and pervasive lay misconceptions about HBV [21]. Not only the knowledge but the researchers also reported that the participants had no access to HBV immunization, testing, and vaccination services. Among barbers in Obuasi municipality, high prevalence (14.5%) of HBV infection was reported [22]. The researchers also reported that 90.5% had heard of HBV but lack knowledge of transmission routes and prevention methods [22]. A similar study among pregnant women attending antenatal care in a mission hospital in Ghana reported 10.2% HBV prevalence, high awareness and poor accurate knowledge about HBV [23].

However, no study on HBV knowledge, vaccination status, perceptions or attitudes was found to have been conducted in the Northern region and more so in the Nanumba North and South districts of the region. No evidence of such a study among young adults in and out of school in the region and the districts is located in the literature. In order to design and implement interventions to prevent spread of infections and to promote voluntary testing and vaccination against HBV, the gap in knowledge must first be bridged. This study was therefore designed to assess rural high school students’ knowledge, perceptions, and vaccination status of HBV in the Nanumba North and South districts.

**Methods:**

We employed proportionate sampling to draw 426 students from Bimbilla Senior High (BSH) in Nanumba North and Wulensi Senior High (WSH) in Nanumba South districts of the Norther Region. We selected proportionate sampling because of the two high schools involved from two districts and needed to have representation of the population sizes of the schools. Proportionate sampling technique is where samples are drawn to be representative of population sub-groups and the sampling fraction in each stratum is made equal to the sampling fraction for the total population [24]. Therefore, 204 students were drawn from BSH and 222 drawn from WSH. All data entry, analysis, and presentation were done using SPSS version 20 graduate pack. Descriptive statistics were used to analyze demographic variables and knowledge based questions. Independent samples *t* test was used to compare means of knowledge scores between males and females and between the two high schools. Multinomial regression was used to develop and test a model for predicting vaccination against HBV. Chi square test of significance was used to test two hypotheses in this study. Ethical review of the study was done and approved by the Ethics Committee of Ghana Health Service (GHSEC) with approval number GHS-ERC:01/05/17 in 2017 and data collected in February, 2018. The district directorates of both Nanumba North and South districts as well as heads of the two high schools gave permission before the study was conducted. Each student was provided written information sheet and informed consent form and had to voluntarily agree or assent to participate in the study before participating in the study.

**Findings:**

Important findings were made in this study and these findings are organized in sub-headings including demographic characteristics, knowledge of hepatitis B, risk of hepatitis B infection, and vaccination status of participants against hepatitis B virus.

**Demographics:**

The participants of the study were high school students sampled from two rural high schools in Nanumba North and South districts of Northern region of Ghana. There were 441 sets of questionnaire completed and received from the students in both Bimbilla Senior High from Nanumba North district and Wulensi Senior High in Nanumba South district. After examining all the questionnaires, 15 questionnaires lacked substantial information and had to be removed from the analysis and presentation. Therefore, 426 questionnaires, above the estimated sample size of 423 were analyzed and included in the results.

The demographic characteristics included in this study were age, sex/gender, and school. Of the 426 students included in the analysis, 204 were from Bimbilla Senior High and 222 from Wulensi Senior High and those constituted 47.9% and 52.1% respectively. The students were made of 270 (63.4%) male and 156 (36.6%) female. The mean age of the students is 18.58 (SD=1.844). The minimum and maximum ages of the 426 students were 13 and 24 respectively.

**Assessment of Knowledge of Hepatitis B Virus:**

In order for individuals to protect themselves against contracting a disease or condition and for those who already contracted a disease to manage the condition effectively, adequate and accurate or comprehensive knowledge of that disease or condition is necessary. We the knowledge of the rural students about hepatitis B virus infection as critical step in the prevention of new infections. In order to do this, we included 16 questions in the questionnaire as knowledge variables. The number of correct answers a participant selected determined how much knowledge she or he possessed about hepatitis B virus. The results are presented in the frequency distribution table one below in relative percentages. The results show average knowledge but not comprehensive knowledge of hepatitis B among the participants in this study. The basic knowledge of hepatitis B was demonstrated in the number and percentage of those who answered the questions correctly. For instance, 295 (69.2%) answered correctly that hepatitis B is caused by a virus while 131 participants answered wrongly by either choosing bacteria or fungus as the source of hepatitis B infection constituting about 30%. Most of the participants also correctly knew that having multiple sexual partners and engaging in unprotected sexual behaviors raised the risk of hepatitis B infection. From the table below, 334(78.4%) and 349 (81.9%) answered correctly that multiple sexual partners and unprotected sexual behavior increased the risk of hepatitis infection respectively. Here again, a significant number of the participants still did not know that having multiple sexual partners and engaging in unprotected sexual intercourse constituting 22% and 18% of the students respectively. Majority of the participants answered correctly hepatitis B can be contracted through transfusion of infected blood, unsafe intravenous drug use, and from pregnant women to their unborn children with 382 (90%), 357 (84%), and 328 (77%) respectively. However, those who did not know that hepatitis B can be contracted through blood transfusion, intravenous drug use, and mother-to-child transmission were 10%, 16%, and 23% respectively. In terms of the body organ affected by hepatitis B viral infection, 396(93%) correctly answered that hepatitis B infection leads to live failure and liver cancer.

Three questions tested the knowledge of the participants on prevention of hepatitis B infection. Participants were asked the best way to protect themselves against hepatitis B infection and the results showed that 344 (81%) correctly chose vaccination with hepatitis B vaccine the best prevention method, 74 (17%) said abstaining from sexual activity, and 2% chose taking medication. The second prevention related question was whether or not participants were aware of availability of hepatitis B vaccine and here again overwhelming majority (90%) knew there is a vaccine to protect against hepatitis B virus. The last prevention related question was how many injections are required of hepatitis B vaccine to be protected. With this question, 70% of the participants did not know and wrongly answered that question. Only 30% correctly answered that three injections are required to be fully protected.

However, majority of the participants also demonstrated high level of misconceptions about hepatitis B virus. For instance, about 46.7% and 54.4% of the 426 participants believed hepatitis B can be contracted through mosquito bites and physical contact such as handshakes and hugging respectively. Moreover, 228 (53.5%) and 325 (76.3%) respectively erroneously believed that hepatitis B is spread through food and breastfeeding.

To further assess the level of knowledge of the participants about hepatitis B, we computed total knowledge score in using SPSS. A score of 14 meant minimal knowledge while a score of 26 indicated comprehensive knowledge of hepatitis B. Descriptive analysis showed a mean knowledge score of 18.4 (SD=2.24). From the above analysis it could be seen clearly that the participants demonstrated general awareness and basic knowledge of hepatitis B viral disease. However, significant numbers still got the answers wrong and with the many misconceptions majority held regarding hepatitis B, comprehensive knowledge of hepatitis B was not demonstrated among the participants in this study.

We also wanted to know if there was significant difference in HBV knowledge between male and female students regarding hepatitis B and between students of BSH and WSH. So we conducted independent-samples T test, a statistic used to determine differences in means of two independent groups. With equal variances assumed, the results showed a mean knowledge score of 18.31 (SD=2.28) and 18.60 (SD=2.17) for males and females respectively. There was no significant difference between males and females regarding knowledge of hepatitis B, with *P-value of 0.20* at 95% confidence interval. There was however, significant difference in HBV knowledge between the two high schools. Independent samples t-test results showed mean knowledge score of 18.91 (SD=2.31) and 17.96 (SD=2.08) for BSH and WSH respectively. With equal variances assumed and at 95% confidence interval, the *P-value* was p=0.000. Therefore, students of BSH significantly possessed higher hepatitis B knowledge than students of WSH in this study. The students of Bimbilla Senior High may have been exposed to some educational program on HBV or some source of information about it.

| Table 1 | | |
| --- | --- | --- |
| *Hepatitis B Knowledge questionnaire responses in relative percentages* | | |
| Question | Frequency | Relative Percent |
| 1. Hepatitis B is a disease caused by |  |  |
| Virus | 295 | 69.2 |
| Bacteria | 102 | 23.9 |
| Fungi | 29 | 6.8 |
| 2. Mosquito bites can give people Hepatitis B |  |  |
| True | 199 | 46.7 |
| False | 227 | 53.3 |
| 3. People can get Hepatitis B through physical contact such as shaking hands and hugging |  |  |
| True | 236 | 55.4 |
| False | 190 | 44.6 |
| 4. Having sexual intercourse with someone who has hepatitis B without condoms can give you hepatitis B |  |  |
| True | 334 | 78.4 |
| False | 92 | 21.6 |
| 5. Having unprotected sex with many people increases the risk for getting infected with hepatitis B virus |  |  |
| True | 349 | 81.9 |
| False | 77 | 18.1 |
| 6. Someone can get hepatitis B through receiving infected blood in his or her body at the hospital |  |  |
| True | 382 | 89.7 |
| False | 44 | 10.3 |
| 7. Someone can get hepatitis B by sharing needles, syringes, and razors with infected persons |  |  |
| True | 357 | 83.8 |
| False | 69 | 16.2 |
| 8. Can a person give hepatitis B to other people but does not know he/she has hepatitis B? |  |  |
| Yes | 357 | 83.8 |
| No | 69 | 16.2 |
| 9. Hepatitis B can be spread through food |  |  |
| True | 228 | 53.5 |
| False | 198 | 46.5 |
| 10. Pregnant women can pass on hepatitis B virus to their newborn babies through birth. |  |  |
| True | 328 | 77 |
| False | 98 | 23 |
| 11. Children can get infected with hepatitis B virus through breastfeeding. |  |  |
| True | 325 | 76.3 |
| False | 101 | 23.7 |
| 12. The best way to find out if you have hepatitis B is through: |  |  |
| Blood test | 354 | 83.1 |
| Urine test | 35 | 8.2 |
| Physical appearance | 37 | 8.7 |
| 13. Hepatitis B is a serious disease that can lead to liver damage, liver failure, liver cancer, or even death. |  |  |
| True | 396 | 93.0 |
| False | 30 | 7.0 |
| 14. The best way to prevent hepatitis B is by: |  |  |
| Getting vaccinated | 344 | 80.8 |
| Abstaining from sex | 74 | 17.4 |
| Taking your medication | 8 | 1.9 |
| 15. There is a vaccine that can protect people against hepatitis B virus |  |  |
| True | 382 | 89.7 |
| False | 44 | 10.3 |
| 16. How many injections do you need to be protected against Hepatitis B? |  |  |
| Correct answer (3) | 127 | 29.8 |
| Wrong answer | 297 | 69.7 |

**Risk of Hepatitis B Virus Infection:**

To examine the risk of hepatitis B infection among the participants in this study, six questions were asked that covered largely behavioral and immune system comprise attributes. This was in addition to age of the participants. The results are presented in table 2 below. The age of the participants is unique and important in this study because of current national policy in Ghana pertaining to immunization against hepatitis virus. In 2003, Ghana Health Service (GHS) introduced a policy to vaccinate all new born babies against hepatitis. It is therefore, assumed that those born after 2003 are protected against the hepatitis B. Prior to 2003, there was no mandatory immunization against hepatitis B and so all those participants who were born before 2003 stood the risk of being infected unless they voluntarily vaccinated themselves against it. Out of the 426 participants, only three (0.7%) were born after 2003 and may have been vaccinated against hepatitis B. The rest were born before 2003 and so faced the risk of infection.

Another risk factor that was considered in this study was whether or not the participants had ever been diagnosed with a sexually transmitted infection (STI) prior to the time of the study. This question was added because research has shown that the presence of existing STI comprises the immune system and increases the risk of other infections. From the table, 62(14.6%.) participants reported that they had been diagnosed with an STI before. Therefore, majority of the participants did not have increased risk of infection from existing STIs. In terms of current risky sexual behavior, participants were asked about the number of sexual partners they had and whether or not they had engaged in unprotected sexual behavior in the last six months before the study. The results show that majority of the students in this study 316(74.2%) reported they had no sexual partners and therefore, assumed not to be sexually active at the time of the study. Students who reported having one sexual partner at the time of the study were 102 which constituted 23.9%. Those who reported having two or more sexual partners were only 8 making up 1.9%. Of the respondents who were sexually active, 86( %) reported of engaging in unprotected sex with their partners, therefore increasing their risk of not just hepatitis B infection but other diseases such as HIV.

Unsafe drug use, especially intravenous drug use, has been linked to hepatitis B infection and so those who engaged in unsafe drug use have increased risk of hepatitis B infection. The question of drug injection was asked in this study and the results also presented in table 2. Out of the 426 participants, 99 (23.2%) students reported that they have injected drugs elevating their risk of hepatitis B infection. Majority (76.8%) however reported never injecting drugs and therefore had lower risk compared to those who reported injecting drugs to hepatitis B infection.

The last two questions we included to examine risk factors of infection were whether had ever been tested for hepatitis B and whether or not they were vaccinated already against hepatitis B. For testing for hepatitis B virus, only 86 (20.2%) reported that they had been tested and so knew their status at the time they were tested. Again, the vast majority 340 (79.8%) reported they had never tested and so did not know their status at the time of this study. On vaccination, a similar trend was found. Only 96 (22.5%) reported they were vaccinated and therefore, protected. Most 330 (77.5%) reported they were not vaccinated and therefore, still faced the risk of infection.

From the above discussion, it is clear that the large majority of the rural students in this study are not protected and faces risk of hepatitis B virus infection.

| Table 2: | | |
| --- | --- | --- |
| Risk Factors for Hepatitis B infection | | |
| Variable | Frequency | Relative percent |
| 1. Have you ever been diagnosed with a sexually transmitted disease |  |  |
| Yes | 62 | 14.6 |
| No | 364 | 85.4 |
| 2. How many sexual partners do you currently have? |  |  |
| None | 316 | 74.2 |
| One | 102 | 23.9 |
| Two or more | 8 | 1.9 |
| 3. Do you inject drugs or have you ever injected drugs? |  |  |
| Yes | 99 | 23.2 |
| No | 327 | 76.8 |
| 4. Have you ever been tested for Hepatitis B? |  |  |
| Yes | 86 | 20.2 |
| No | 340 | 79.8 |
| 5. In the last 6 months have engaged in unprotected sexual behavior? |  |  |
| Yes | 86 | 20.2 |
| No | 340 | 79.8 |
| 6. Have you vaccinated yourself against Hepatitis B? |  |  |
| Yes | 96 | 22.5 |
| No | 330 | 77.5 |

**Vaccination Status of Participants:**

One of the goals of this study was to determine the vaccination status of the rural students against HBV. The key condition we asked to determine the vaccination status was whether or not they ever received or at least recall being vaccinated against hepatitis B virus. From the results, majority of the participants reported not being vaccinated against hepatitis B at the time study was conducted. Out the 426 students whose questionnaires were analyzed, only 86 (21%) reported that they tested for HBV.However, in total, 96 participants reported being vaccinated against hepatitis B virus and this constituted 22.5%.. It is therefore not out of place to state that the students were largely not protected against hepatitis B infection and that majority of the students did not know their status with regards to HBV.

We developed a model using multiple regression with vaccination against hepatitis B virus being the outcome variable. The predictor variables we included were previous testing for hepatitis B virus, gender, knowledge score, number of sexual partners, whether or not the participant is ever diagnosed with a sexually transmitted disease, and high school the student came from. The model showed that only previously being tested for hepatitis B virus significantly predicted vaccination against hepatitis B virus infection with P-value of P=0.000 at 95% confidence interval.

**Previous HBV testing and its correlates**

We tested two hypotheses in this study

1. There is no significant association between knowledge of hepatitis B virus and testing to know one’s status of hepatitis B among the rural high school students.
2. There is no significant relationship between testing to know one’s hepatitis B status and vaccination against hepatitis B.

For the first hypothesis, Pearson X^2^ of association showed no significant association between knowledge of HBV and taking HBV test to know one’s status among the rural students who participated in this study. The results showed *X^2^(9.705)=12, p=0.642* at 95% confidence interval. We therefore, failed to reject the null hypothesis that there is no significant association between knowledge of HBV and taking HBV test. On the other hand, the result of the second hypothesis showed significant association between taking HBV test and vaccinating oneself against its infection. Pearson X^2^ showed *X^2^(25.910) =1, P=	0.000* at 95% confidence interval indicating significant association. We, therefore, reject the null hypothesis that there is no significant association between taking HBV test and vaccinating against the HBV infection.

**Discussion:**

Knowledge of Hepatitis B: The results presented from study above show basic but not adequate or comprehensive knowledge of hepatitis B virus, its modes of infection, and prevention of hepatitis B. The finding of basic awareness rather than comprehensive knowledge of HBV in this study is consistent with similar findings in Ghana and other sub-Saharan African countries. For instance, among 175 healthcare workers at a public hospital in ….Ghana, knowledge about HBV was basic and comprehensive knowledge even among the healthcare workers was poor [21]. Again, among 200 barbers in Obuasi in the Ashanti region of Ghana, 90.5% lacked knowledge about HBV [22]. In Nigeria, similar study among 643 pregnant women found that 76% reported poor knowledge of HBV. Studies in other parts of the world have reported similar trend of poor knowledge [15, 16].. In this study, demographic variables such as age and sex had no correlation with knowledge of HBV. The school the student attended was the only variable that significantly predicted knowledge of HBV in that students of Bimbilla Senior High reported significantly higher knowledge than students of Wulensi Senior High. The difference in knowledge may be the result of exposure to information or educational program of students of Bimbilla Senior High.

Risk of Infection: The findings show that majority of the students were not sexually active at the time of the study and therefore, most did not engage in risky sexual behavior. However, the risk of infection of hepatitis B in this population was still high because majority had not been tested and vaccinated against HBV. This is because out of the 426 students, only 86 (20%) reported that they ever screened for HBV while 80% never screened and yet most did not know their status with regards to HBV. This finding is also similar with the pattern revealed with the existing literature as many studies have reported low screening. For example, among 9,014 adults sampled from the general population in France, it was reported that only 27% ever screened [16]. In the study among pregnant women cited earlier, only 20% reported ever screening for HBV [15].

Another important finding in this study was the high prevalence of HBV self-reported by the participants. Out of the 86 students who reported being screened for HBV, 39 stated they tested positive and were HBV patients and this constituted 45.3% of those who screened. This is about four times of the 12% hepatitis B national prevalence rate reported and many other rates reported across Ghana and other countries. For instance, 10.8% and 11.6% prevalence was reported among 576 voluntary blood donors and blood replacement donors respectively in Tamale, the Northern regional capital in Ghana [4]. In the Eastern region, 10.6% prevalence was reported among 1500 pregnant women [5]. It is also higher than prevalence rates reported in Ghana [14, 22, 23].

The ultimate goal of the fight against HBV is to prevent new infections and the most effective means of achieving zero infections is vaccination against the virus. However, the findings in this study is that there is low vaccination among the population and this may be reflection of the true situation in the rural districts. From the results above, only 96 of the 426 students reported that they were vaccinated which is just 22.5% in this study.

Finally, testing for HBV was found to be significantly associated with being vaccinated against the virus. This underscores the importance of HBV screening programs as a critical strategy to motivating vaccination and reducing infections.

**Conclusion:**

While we exercise caution in generalizing the results of this study to all rural high school students in Ghana, the results of this study has re-echoed the high prevalence of HBV in Ghana. The poor state of knowledge and high risk of HBV infection among young adults in rural communities have also been highlighted in the findings of this study. The results have also shown that vaccination against HBV infection is low among rural students and young adults at least in the Nanumba North and South districts of Ghana. Finally, HBV screening is shown to be significantly associated with vaccination against the virus. There is need for culturally appropriate and evidenced-based educational interventions to improve knowledge of HBV. Developing and implementing a national HBV screening and vaccination programs is critical in winning the fight against the increasing morbidity and mortality caused by HBV infection in Ghana.

**References:**

1. CDC. Hepatitis B. 2019. Retrieved from <https://www.cdc.gov/hepatitis/hbv/index.htm>
2. World Health Organization. Hepatitis B. 2019. Retrieved from <https://www.who.int/en/news-room/fact-sheets/detail/hepatitis->b
3. Kew MC. Epidemiology of chronic hepatitis B virus infection, hepatocellular carcinoma, and hepatitis B virus-induced hepatocellular carcinoma. Pathologie Biologie. 2010 Aug 1;58(4):273-7.
4. Dongdem JT, Kampo S, Soyiri IN, Asebga PN, Ziem JB, Sagoe K. Prevalence of hepatitis B virus infection among blood donors at the Tamale Teaching Hospital, Ghana (2009). BMC research notes. 2012 Dec;5(1):115.
5. Cho Y, Bonsu G, Akoto-Ampaw A, Nkrumah-Mills G, Nimo JJ, Park JK, Ki M. The prevalence and risk factors for hepatitis B surface Ag positivity in pregnant women in eastern region of Ghana. Gut and liver. 2012 Apr;6(2):235.
6. Geretti AM, Patel M, Sarfo FS, Chadwick D, Verheyen J, Fraune M, Garcia A, Phillips RO. Detection of highly prevalent hepatitis B virus coinfection among HIV-seropositive persons in Ghana. Journal of clinical microbiology. 2010 Sep 1;48(9):3223-30.
7. de Mendoza C, Bautista JM, Pérez-Benavente S, Kwawu R, Fobil J, Soriano V, Díez A. Screening for retroviruses and hepatitis viruses using dried blood spots reveals a high prevalence of occult hepatitis B in Ghana. Therapeutic advances in infectious disease. 2019 May;6:2049936119851464.
8. GHS DHIMS
9. CDC. Viral hepatitis B information: perinatal transmission. 2015. Retrieved from

<http://www.cdc.gov/hepatitis/hbv/perinatalxmtn.htm>

10. Pham DA, Leuangwutiwong P, Jittmittraphap A, Luplertlop N, Bach HK, Akkarathamrongsin S, Theamboonlers A, Poovorawan Y. High prevalence of Hepatitis C virus genotype 6 in Vietnam. Asian Pacific journal of allergy and immunology. 2009 Jun 1;27(2-3):153.
11. Agyeman AA, Ofori-Asenso R. Prevalence of HIV and hepatitis B coinfection in Ghana: a systematic review and meta-analysis. AIDS research and therapy. 2016 Dec;13(1):23.
12. Owusu M, Bonney JK, Annan AA, Mawuli G, Okyere K, Mutocheluh M, Aryeequaye J, Adjei NK, Afihene M, Spangenberg K, Sylverken J. Aetiology of viral hepatitis among jaundiced patients presenting to a tertiary hospital in Ghana. PloS one. 2018 Sep 12;13(9):e0203699.
13. Adeyemi AB, Enabor OO, Ugwu IA, Bello FA, Olayemi OO. Knowledge of hepatitis B virus infection, access to screening and vaccination among pregnant women in Ibadan, Nigeria. Journal of obstetrics and gynaecology. 2013 Feb 1;33(2):155-9.
14. Hagan OC, Nsiah P, Obiri-Yeboah D, Yirdong F, Annan I, Eliason S, Nuvor SV. Impact of universal childhood vaccination against hepatitis B in Ghana: A pilot study. Journal of public health in Africa. 2018 Oct 1;9(2).
15. Abeje G, Azage M. Hepatitis B vaccine knowledge and vaccination status among health care workers of Bahir Dar City Administration, Northwest Ethiopia: a cross sectional study. BMC infectious diseases. 2015 Dec;15(1):30.
16. Brouard C, Gautier A, Saboni L, Jestin C, Semaille C, Beltzer N. Hepatitis B knowledge, perceptions and practices in the French general population: the room for improvement. BMC public health. 2013 Dec;13(1):576.
17. Abbasi I, Fatmi Z, Kadir M, Sathiakumar N. Prevalence of hepatitis B virus infection among barbers and their knowledge, attitude and practices in the district of Sukkur, Sindh. International journal of occupational medicine and environmental health. 2014 Oct 1;27(5):757-65.
18. Carabez RM, Swanner JA, Yoo GJ, Ho M. Knowledge and fears among Asian Americans chronically infected with hepatitis B. Journal of Cancer Education. 2014 Sep 1;29(3):522-8.
19. Afihene MY, Duduyemi BM, Hannah-Lisa A, Khatib M. Knowledge, attitude and practices concerning Hepatitis B infection, among healthcare workers in Bantama, Ghana: a cross sectional study. International Journal of Community Medicine and Public Health. 2017 Feb 5;2(3):244-53.
20. Adekanle O, Ndububa DA, Olowookere SA, Ijarotimi O, Ijadunola KT. Knowledge of hepatitis B virus infection, immunization with hepatitis B vaccine, risk perception, and challenges to control hepatitis among hospital workers in a Nigerian tertiary hospital. Hepatitis research and treatment. 2015;2015.
21. Mkandawire P, Richmond C, Dixon J, Luginaah IN, Tobias J. Hepatitis B in Ghana's upper west region: a hidden epidemic in need of national policy attention. Health & place. 2013 Sep 1;23:89-96.
22. Adoba P, Boadu SK, Agbodzakey H, Somuah D, Ephraim RK, Odame EA. High prevalence of hepatitis B and poor knowledge on hepatitis B and C viral infections among barbers: a cross-sectional study of the Obuasi municipality, Ghana. BMC public health. 2015 Dec;15(1):1041.
23. Adjei CA, Atibila F, Apiribu F, Ahordzor F, Attafuah PA, Ansah-Nyarko M, Asamoah R, Menkah W. Hepatitis B Infection among Parturient Women in Peri-Urban Ghana. The American journal of tropical medicine and hygiene. 2018 Dec 5;99(6):1469-74.
24. Fink A. How to sample in surveys. Sage; 2003.
