# Supplementary material for: Knowledge, risk of infection, and vaccination status of hepatitis B virus among rural high school students in Nanumba North and South Districts of Ghana
Source: PLoS One. 2020 Apr 22;15(4):e0231930. doi: 10.1371/journal.pone.0231930 (PMC7176126; doi:10.1371/journal.pone.0231930)
Supplement: Attachment — Submitted filename: Response to Reviewers HBV Knowledge.docx [file pone.0231930.s002.docx]

**Response to Reviewers HBV Knowledge**

The following are responses on how we have addressed the comments and recommendations from the reviewers.

1. Ghana is part of SSA.

**Response: Removed**

2. Do you mean research?

**Response: Yes, we meant research. Changed**

3. Not necessary: Response:

**Statement removed from abstract.**

4. This, alone, does not pass any information across. What is the maximum obtainable score?

**Response: The minimum score was 3 and the maximum score was 16. This has been clarified in the abstract. Lines 46-47.**

5. Do you mean ‘dependent’? **Response: Yes. Changed.**
6. This is unclear. What do you mean when you say the data is ‘scary’. How does one measure if attention to a disease is adequate. You might want to include a reference here or add some text to buttress the facts in this sentence.

**Response: The sentence has been reconstructed and reference added. Lines 72-76**

7. This should be written as sub-Saharan Africa with an ‘n’.

**Response: This is corrected accordingly.**

8. This is unclear. What do you mean by ‘global picture’, do you mean to say ‘global problem’? I suggest you reword the sentence.

**Response: The sentence has been restated differently.**

9. Please insert reference here.

**Response: Reference added. Line 87 on page 4.**

10. This sentence seems out of place here. You should consider moving it upwards with the sentence stating that 2 billion persons globally have been infected by HepB.

**Response: The sentence has been moved up as suggested. Lines 84-85**

11. Inconsistency with formatting here.

**Response: Formatting corrected. Lines 89-91**

12. This section needs major work. You might want to consider having subheadings under this section. Each subhead would address the methods for each key component of your study. Sample headings could be Sampling strategy, knowledge assessment, assessment of HBV infection risk etc. You already have a lot of the methods in other sections. Just rewrite them for readability and reproducibility then include them in this section. I have included some comments in the different sections that you might want to consider in reworking this section

**Response: The whole methods section is reorganized and put into subheadings for clarity. This can be found on pages 9-13 and from line 203 to line 295.**

13. What type of regression (logistic or linear)

**Response: Logistic regression. Clarified.**

14. A major assumption for using t. test is normality of dependent variable. Did you test for normality of the dependent variable? I suspect that the distribution of the dependent variable would be skewed. If this is the case, I suggest you use non-parametric alternatives such as Mann-Whitney test instead.

**Response: We performed a normality test and realized the distribution was skewed. Based on your recommendation, we changed the statistic and performed a Mann-Whitney U test instead. This is stated in the methods section and presented in the findings section respectively.**

15. It would be better to place the percentages beside the figures like so 204 (47.9%).

**Response: this has been corrected throughout.**

16. Ensure to use reported speech. This should be ‘was’.

**Response: This has been corrected according throughout the paper.**

17. Not necessary. **Response: statement removed.**
18. Not clear: **Response: A word was missing that made the sentence vague. The word ‘view’ has been inserted and the sentence reads correct and understandable. Line 317 in bold.**
19. We could have a table of the sample characteristics in relation to knowledge, risk of infection and vaccination status if the data is available.

**Response: We felt that only three demographic variables including age, gender/sex, and school were included and the data presented. The results in relation to knowledge of HBV and risk of infection are already presented in tables 1 and 3 respectively.**

20. What % of response rate was that?

**Response: This is clarified and on lines 303-307.**

21. Did you check for the reliability of this knowledge scale?

**Response: No. We developed the questionnaire based on CDC’s information on HBV and other sources and pre-tested the instrument prior to collecting data.**

22. This is better written as 1. **Response: corrected.**
23. How did you determine what average and comprehensive knowledge is?

**Response: For the purpose of this study we defined HBV knowledge which is clarified in the methods section. Based on your recommendation we categorized knowledge into poor, basic, and good. This can be found on pages 9-10 and lines 214-221**

24. Perhaps a scaler kind of measurement to distinguish the different levels of knowledge e.g. comprehensive-scoring 12/16, average scoring 9/16 and basic anything between 1-8?

**Response: A scale has been developed. Poor knowledge (1-5 correct answers), basic ( 6-12 correct answers), good knowledge (13-16 correct answers). This is presented on lines 217-220.**

25. It is not standard writing practice to repeat information already included in a table. I suggest you rewrite this entire section to highlight the most important findings:

**Response: Based on your recommendation we have rewritten the section into brief summary and referred readers to the table. We have also changed the statistic to Mann-Whitney test. This whole section can be found on pages 15-17 and lines 325-359**

26. I think we can loss all these once we refer readers to the table. This can then be elaborated on in the discussion section.

**Response: The whole section was taken out of the manuscript as described in comment 28 above.**

27. You stated earlier that 16 questions were used in the knowledge assessment. How did you score these questions? Did you assign 2 points to a correct response and zero points to an incorrect response? I suggest you properly highlight the calculations for computing this score. What are the minimum and maximum obtainable scores? Is there a rationale for choosing 14 and 26 as thresholds for minimal and comprehensive knowledge? Finally, I suggest you consider using other words than ‘minimal’ and ‘comprehensive’. Maybe use ‘poor’ and ‘good’ knowledge. If you choose to retain these words, please define them as used in the context of this study. **Response: We took your recommendation and defined the parameters accordingly using poor, basic, and good knowledge.**
28. What qualifies as basic knowledge? Could you also include the minimum and maximum score obtained in this study to maybe give some context to your reader. **Response: Addressed.**
29. Here we could argue that being aware of the virus was critical to having either comprehensive, moderate or basic knowledge of the disease. We, however, have to define the parameters of the different levels of knowledge and what each entailed. **Response: addressed as stated in 30 above.**
30. This is a bit confusing. Were you measuring awareness or knowledge? What do you mean by general awareness? Could these two constructs be proxies?

**Response: We measured knowledge but in the categories we created, awareness falls in poor knowledge. This has been address as indicated above.**

31. I think you have already established this in a previous paragraph.

**Response: Statement removed**

32. This might not be necessary. When you don’t assume equal variances, it’s called a Welch’s test. Moreso did you check for equality of variance before deciding on this assumption?

**Response: Different statistic used as recommended and so statement removed.**

33. This should be in the methods section. Just state that all statistically analysis was conducted at 95% interval. **Response: this is removed and clarified in methods section on line 283.**
34. Interesting results. This might not be the best way to report these results. You could try something like this. *Female respondents had higher knowledge scores than their male counterparts however the difference was not statistically significant (p = 0.20)* You could also check this link for how to report t-test results in APA format <https://statistics.laerd.com/spss-tutorials/independent-t-test-using-spss-statistics.php> It might also be a good idea to check if knowledge scores differ by gender while controlling for school. i.e. do the test in each school. **Response: Mann Whitney U test used instead of t-test and as such the results has changed.**
35. This should be in the discussion section. I know you used proportionate sampling, but could this maybe be as a result of oversampling students in STEM in BSH as compared to WSH. Is there a relationship between knowledge and age? Maybe do a correlation (spearman or pearson’s). Alternatively, you could create age bins and then do an ANOVA(Parametric) or Kruskal Wallis(non-parametric) test.

**Response: The statement has been removed due to the change in analysis from t test to Mann-Whitney test. We have also performed Pearson’s correlation and found no correlation between age and HBV knowledge. This is on lines 356-359.**

36. Do you mean risk factors? You should rework this section for readability.

**Response: The title is changed to assessment of risk of HBV infection and on line 361**

37. Does this not fall under biological? **Response: Yes, it does. Removed**
38. This should go in your introduction.

**Response: The statement moved to introduction lines 140-142**

39. This should go in your methods section.

**Response: Statement move to methods section lines 271-273.**

40. This could go into the introduction section or form part of your discussion.

**Response: The statement is moved to discussion and can be seen on lines 462-465.**

41. You did not assess this?

**Response: We assessed and the statement is corrected and on line 361.**

42. This is only an assumption. You have data on vaccination and the data does not support this assertion.

**Response: Yes, it was an assumption we made. Statement rephrased. LINE 366**

43. Do you mean compromises? You also add a reference.

**Response: Yes, compromises. Corrected. Reference added. Line 364-366.**

44. What table? **Response: Table number added**
45. Make similar changes through out the manuscript.

**Responses: Addressed**

46. This is confusing. In a previous sentence, you stated that the presence of an STI predisposes to HBV infection. You state here that participants stated they had been previously diagnosed with an STI. Does a previous diagnosis also predispose to HBV infection?

**Response: It is the wording in the write up that may have created the confusion. Corrected. The participants indicated they had been diagnosed in the past six months.** **Line 366-367.**

47. Is this necessary? You don’t need to state all results if they are already in a table.

**Response: Phrase removed.**

48. I suggest you delete this unless you can confirm from literature that previous STI predisposes to HBV. I have not looked at the literature, but I suspect that there is no direct relationship. **Response: Statement deleted as recommended.**
49. This denominator for this percentage should be the total number of persons who were sexually active. Is having one sexual partner a risk factor for HBV infection? You might also want to check if there is a relationship between those who engage in unprotected sexual activity and previous diagnosis of STI. **Response: Corrected and recalculated.** **Lines 374.**
50. Introduction.

**Response: Statement moved to introduction lines 124-126**

51. This question may be misleading to a respondent. The respondent could interpret this as receiving injections from a healthcare provider.

**Response: The question was clear to the students on the actual questionnaire as narcotic or illegal drugs as commonly known in Ghana was clarified. It was in the SPSS variable labeling that the narcotic/illegal was left out. This is corrected in the table 3.**

52. You might want to reconsider your previous statement about vaccination pre and post the 2003 government decree.

**Response: Statement corrected on line 361.**

53. Wouldn’t this be better as part of your discussion?

**Response: Statement moved to discussion. Lines 452-454.**

54. Move to methods. **Response: Statement moved to methods as recommended.**
55. Include the regression table with coefficients, p values and reference groups. Did you convert categorical variables to dummy or indicator variables? **Regression table included page 21**
56. I almost never see authors report hypothesis testing this way. I suggest you just state the results without talking about rejecting the null or otherwise. Maybe just remove the hypotheses all together and state the results of the chi-square under this section.

**Response: Recommendation followed and whole paragraph removed and a statement added on lines 403-405**

57. What are the implications for some of your findings to the study location and Ghana? Could you maybe relate your findings to bigger goals such as the SDGs i.e. what is the target for the SDGs and how effective are the current strategies. What needs to be done to cover up any gaps if gaps exist.

**Response: We have looked and made inference with regards to SDGs and this can be found on lines 455-457**

58. You seem to be missing something here. **Response: addressed.**
59. Unclear: **Response: Clarified**
60. This correlation with age is not shown in your results. **Response: This is indicated on lines 353-354**
61. This is repeated in the results section. Delete this from the results section**. Response: Deleted as statistic changed.**
62. Can the first part of the date presentation look at the demographic variables and the key objectives of the study?

**Response: We actually started presenting the findings with the paragraph on demographic variables and have realigned them following your recommendation.**

63. This should be in the discussion section. I know you used proportionate sampling, but could this maybe be as a result of oversampling students in STEM in BSH as compared to WSH. Is there a relationship between knowledge and age? Maybe do a correlation (spearman or pearson’s). Alternatively, you could create age bins and then do an ANOVA(Parametric) or Kruskal Wallis(non-parametric) test.

**Response: We have performed Pearson correlation coefficient to examine knowledge of HBV and age. Lines 353-354**

64. This was not presented in the results.

**Response: We have presented this in the Risk of infection section. Lines 385-387**

65. This does not sound right. Did you use the same denominator as these figures you are comparing with? The correct rate should be around 9%.

**Response: It is true that it is 9% when you consider the total sample of 426 in the study. We initially calculated based the 86 students who reported being tested for HBV. We have corrected it to 9% and this can be found on lines 385-387**

66. See previous comment. **Response: Addressed** .
67. What’s the implication of this?

**Response: Implication for Ghana has been added and on lines 455-457**
