# Supplementary material for: Knowledge, risk of infection, and vaccination status of hepatitis B virus among rural high school students in Nanumba North and South Districts of Ghana
Source: PLoS One. 2020 Apr 22;15(4):e0231930. doi: 10.1371/journal.pone.0231930 (PMC7176126; doi:10.1371/journal.pone.0231930)
Supplement: Attachment — Submitted filename: Response to reviewers HBV.docx [file pone.0231930.s004.docx]

**Response to reviewers’ comments HBV Manuscript**

The following are the comments made by reviewers and how we addressed them.

1. Table 3?

**Response: Yes, table three. Corrected and on line 214.**

2. Move to the methods section.

**Response: The statement has been moved to methods section and on lines 207 to 213.**

3. Consider removing the valid percent and cumulative percent columns

**Response: We have removed the valid and cumulative percent columns as recommended. Page 16.**

4. What was the correlation coefficient? While the correlation might not be statistically significant, it is important to still report the actual correlation coefficient

**Response: The correlation coefficient has been added (*r*(424)= 0.034, *p*= 0.486) and on line 346.**

5. Please indicate what each variable is i.e q2,q3 etc

**Response: We have indicated what the variables presented in the regression table are and the list is on lines 400 to 402.**

6. Incomplete reference

**Response: A complete reference is provided. Reference number 8.**

7. Country and page numbers missing

**Response: Country and page numbers added. Reference number 25.**

8. Incomplete reference

**Response: Complete reference provided. Reference number 27.**
